# Regulation of ABCC6 Trafficking and Stability by a Conserved C-terminal PDZ-Like Sequence

**DOI:** 10.1371/journal.pone.0097360

**Published:** 2014-05-19

**Authors:** Peng Xue, Chelsea M. Crum, Patrick H. Thibodeau

**Affiliations:** Department of Cell Biology, University of Pittsburgh School of Medicine, Pittsburgh, Pennsylvania, United States of America; Harvard Medical School, United States of America

## Abstract

Mutations in the ABCC6 ABC-transporter are causative of pseudoxanthoma elasticum (PXE). The loss of functional ABCC6 protein in the basolateral membrane of the kidney and liver is putatively associated with altered secretion of a circulatory factor. As a result, systemic changes in elastic tissues are caused by progressive mineralization and degradation of elastic fibers. Premature arteriosclerosis, loss of skin and vascular tone, and a progressive loss of vision result from this ectopic mineralization. However, the identity of the circulatory factor and the specific role of ABCC6 in disease pathophysiology are not known. Though recessive loss-of-function alleles are associated with alterations in ABCC6 expression and function, the molecular pathologies associated with the majority of PXE-causing mutations are also not known. Sequence analysis of orthologous ABCC6 proteins indicates the C-terminal sequences are highly conserved and share high similarity to the PDZ sequences found in other ABCC subfamily members. Genetic testing of PXE patients suggests that at least one disease-causing mutation is located in a PDZ-like sequence at the extreme C-terminus of the ABCC6 protein. To evaluate the role of this C-terminal sequence in the biosynthesis and trafficking of ABCC6, a series of mutations were utilized to probe changes in ABCC6 biosynthesis, membrane stability and turnover. Removal of this PDZ-like sequence resulted in decreased steady-state ABCC6 levels, decreased cell surface expression and stability, and mislocalization of the ABCC6 protein in polarized cells. These data suggest that the conserved, PDZ-like sequence promotes the proper biosynthesis and trafficking of the ABCC6 protein.

## Introduction

Pseudoxanthoma elasticum (PXE) is a disease characterized by the progressive mineralization of elastic fibers. [1], [2] The mineralization and eventual degradation of these fibers cause a loss of elasticity in a variety of affected tissues. While the molecular mechanisms leading to the mineralization processes are unknown, mutations in the ABCC6 ATP-binding cassette (ABC-) transporter have been shown to be causative of the disease. [3], [4] To date, more than 250 coding and noncoding mutations have been identified in *ABCC6* that are associated with PXE. [5]–[7] The resulting loss of protein function in the basolateral membrane putatively alters the secretion of one or more unknown circulatory factors that systemically affect the mineralization of elastic fibers. [8] This mineralization and subsequent degradation of elastic fibers lead to loss of vascular tone, premature arteriosclerosis, laxity in the skin, and loss of vision, resulting from neovasculariziation in the eye. [2].

The ABC-transporter family of proteins is responsible for the secretion of a variety of biological molecules across the cell membrane in an ATP dependent manner. [9] Structurally, the proteins are composed of at least four core domains: two transmembrane domains (TMDs) and two nucleotide-binding domains (NBDs). ATP binding between the NBDs induces their dimerization, which, in turn leads to ATP hydrolysis. [10]–[12] These ATP-induced conformational changes are coupled through a conserved interface to the TMDs, which utilize the energy of ATP binding and hydrolysis to facilitate solute transport. [13], [14] In addition, the long form ABCC subfamily members, including ABCC6, contain an additional N-terminal transmembrane domain whose function is not well defined. [9] Alterations in protein biosynthesis, protein trafficking and localization, ATP binding and hydrolysis, and solute recognition and binding have all been implicated as molecular pathologies associated with ABC-transporter mutations [15]–[18].

The trafficking of multiple ABC transporters is regulated, in part, by C-terminal PDZ (PSD95/Dlg/ZO-1) ligands. [19]–[24] The short C-terminal peptide sequences are bound by PDZ domain-containing proteins. These multi-domain proteins facilitate protein-protein interactions by acting as scaffolds, binding their respective PDZ ligands and holding their partners in close physical proximity. These associations have been shown to regulate protein activity, protein stability and protein mobility in the membrane. [22]–[25] Multiple modes of peptide binding have been ascribed to different classes of PDZ domains. [26] Specificity for these interactions is thought to come from both the sequences of the different ligands and subcellular localization of their PDZ-domain containing protein partners. Within the ABCC subfamily of human ABC-transporters, multiple PDZ ligands have been identified and characterized. Alteration to these sequences results in mislocalization, reduced stability and increased mobility in other members of the ABCC subfamily, including the multi-drug transporters and CFTR [22], [25], [27]–[29].

To evaluate the role of the C-terminal sequence from ABCC6, mutations to the PDZ-like sequence were generated and protein biosynthesis, trafficking and turnover were analyzed in both non-polarized and polarized cells. Deletion of the C-terminal six amino acids, which constitute a PDZ-like ligand sequence, resulted in a significant decrease in steady state levels of ABCC6. These changes were the result of mislocalization within the cell and an increase in protein degradation. In polarized cells, deletion of the C-terminal residues resulted in inefficient targeting to the basolateral membrane. These data demonstrate that the non-canonical PDZ-like sequence found at the C-terminus of ABCC6 contributes to its regulation in the cell. Characterization of the trafficking and regulation of ABCC6 by its physiological binding partners will provide additional insight into the molecular regulation of ABCC6 in normal physiology and the pathophysiology associated with PXE.

## Materials and Methods

### Cloning and Site-directed Mutagenesis

The full-length ABCC6 open reading frame was cloned into pcDNA3.1 (Invitrogen) for CMV-driven expression in mammalian cells. PCR-based site directed mutagenesis was used to introduce specific mutations in the pcDNA-ABCC6 plasmid (QuikChange, Stratagene). The BLAP tagged ABCC6 protein was constructed by PCR insertion of the acceptor peptide sequence (GLNDIFEAQKIEWHE) after Pro4 in the native ABCC6 sequence in the pcDNA3.1 vector. The pBUDD-BirA-KDEL plasmid was a generous gift from Dr. Daniel C. Devor (University of Pittsburgh). Site directed mutagenesis and full-length sequences were confirmed by automated DNA sequencing.

### Cell Culture and Western Blotting

HEK and MDCK cells were obtained from ATCC. HEK293 cells were routinely maintained in DMEM (Gibco) supplemented with 10% v/v FBS, 100 units/ml penicillin and 100 µg/ml streptomycin at 37°C in a 5% CO_2_ environment. MDCK cells were routinely maintained in αMEM (Gibco) supplemented with 10% v/v FBS, 100 units/ml penicillin and 100 µg/ml streptomycin at 37°C in a 5% CO_2_ environment. Cells were transfected using either Fugene6 (Promega) or XtremeGene9 (Roche) lipid transfection reagents. For HEK293 cells, cells were transfected following manufacturers’ protocols and lysed 48–72 hours post-transfection for analysis. Cell surface biotinylation experiments were performed using Sulfo-NHS-Biotin (Pierce) following manufacturers’ protocols. For expression in MDCK cells, cells were transfected at 80% confluence on 0.2 micron Transwell inserts (Costar). Cells were allowed to polarize for 4 days prior to analysis. Polarization was confirmed by staining with the tight junction marker ZO-1. For western blotting, cells were lysed in RIPA (Millipore) and lysates were cleared by centrifugation at 21,000 G for 10 minutes at 4°C. Lysates were separated by SDS-PAGE on Tris-glycine gels and transferred to PVDF membrane. At least 3 independent experiments were performed for each analysis and representative western blots are shown.

### Antibodies and Labeling Reagents

Western blotting and immunofluorescence for ABCC6 were performed using the rat monoclonal α-ABCC6 antibody M6II-7 or M6II-31 (Santa Cruz), which were raised against a portion of TMD2. Polyclonal α-ZO1 antibody (Cell Signalling) was used in immunofluorescence to confirm polarization of the MDCK cells. A mouse monoclonal α-tubulin antibody (Sigma-Aldrich) or α-PARP1 (GeneTex) were used for loading controls. AlexaFluor488 and 555-conjugated streptavidin were used to label the BLAP-containing ABCC6 constructs at the cell surface (Invitrogen). Phalloidin and WGA (Invitrogen) were used as cell surface markers; DAPI (Sigma-Aldrich) was used to stain nuclei.

### Immunofluorescence, Fluorescence Labeling and Fluorescent Microscopy

HEK293 and MDCK cells expressing the pcDNA-ABCC6 proteins were utilized for immunofluorescence. Following expression, cells were washed three times in PBS at 4°C and fixed using 2% PFA in PBS for 10 minutes at 4°C. Cells were blocked with BSA and stained using the antibodies described above. AlexaFluor-conjugated secondary antibodies were used to visualize the ABCC6 and ZO1 proteins (Invitrogen). Immunofluorescence was visualized on either an Olympus IX81 fluorescence microscope or a Fluoview 1000 confocal microscope. Immunofluorescence images were collected from multiple fields from at least four independent experiments. At least 16 fields were evaluated for each of the conditions evaluated. Blind experiments were performed to validate the differences in observed trafficking and expression of the various ABCC6 constructs.

The pcDNA-BLAP-ABCC6 protein was utilized to evaluate cell surface ABCC6. HEK293 or MDCK cells were seeded onto glass coverslips pretreated with poly-lysine (Sigma). Cells were co-transfected with the pcDNA-BLAP-ABCC6 plasmid and the pBUDD-BirA-KDEL plasmid and proteins were allowed to express for 24–48 hours in HEK293 cells and for 5 days in MDCK cells. Following expression, the media was aspirated from the cells and the cells were washed with PBS with 2% BSA on ice. Alexafluor conjugated streptavidin was incubated with the cells in a solution of PBS and 2% BSA for 10 minutes on ice. The labeling mix was aspirated and the cells were washed three times with PBS to remove unbound streptavidin. The cells were either fixed with 2% PFA in PBS or returned to the CO_2_ incubator for kinetic experiments and fixed after specific incubation periods.

### NBD Protein Expression and Analysis

The boundaries of the NBD2 domain were predicted utilizing multiple sequence alignments and structural analysis of extant NBD crystal structures. The predicted coding sequence was PCR amplified and cloned into the pSmt3 vector, which includes an N-terminal 6x His tag and the yeast SUMO protein, Smt3, to aid in solubility. [30] Expression and purification followed procedures previously described for the NBDs from CFTR. [31], [32] *E coli* BL21 (DE3) cells were transformed with the expression plasmids and single colonies were picked to grow an inoculum overnight at 37°C. A one liter expression culture of either the wildtype or mutant NBD2 was inoculated and the cultures were grown at 37°C until an OD_600_ of ∼1.0 in LB broth. Protein expression was induced with 1 mM IPTG and the cultures were shifted to 15°C for expression overnight. Cells were harvested by centrifugation and lysed by sonication in ice cold binding buffer (50 m Tris, 150 mM NaCl, 10% w/v glycerol, pH 7.6) with 2 mM ATP and 1 mM beta-mercaptoethanol. The lysate was clarified by centrifugation (40,000 G RCF, 4°C, 40 minutes) and the soluble fraction was loaded onto a Ni-NTA column (GE HealthSciences) pre-equilibrated in binding buffer lacking ATP. The protein was washed and eluted in binding buffer supplemented with 60 or 400 mM imidazole, respectively. Fractions were collected and samples containing NBD2 protein were immediately supplemented with 2 mM ATP and 1 mM DTT (final concentrations). The fractions were pooled and loaded onto a HiPrep Sephacryl S-300 gel filtration column (GE HealthSciences) equilibrated with binding buffer supplemented with 2 mM ATP and 1 mM DTT. Fractions containing NBD2 were pooled and digested with Ulp1 to liberate the N-terminal His-Smt3 tags and the protein was applied to a Ni-NTA column equilibrated with binding buffer supplemented with 2 mM ATP and 1 mM DTT. The Ulp1 and Smt3 proteins bound to the Ni-NTA column via their respective His tags and the flow through containing NBD2 as collected, concentrated and stored at −80°C prior to use. Circular dichroism (CD) spectroscopy and analytical gel filtration chromatography (GFC) were performed to assess changes in secondary structure and hydrodynamic radius. CD spectra were collected on a Jasco 810 spectropolarimeter, as previously described. [33] Analytical GFC was performed using a Superdex S75 10/300 GL (GE HealthSciences) column to evaluate changes in hydrodynamic radius.

## Results

### C-terminally Regulated Trafficking of the ABCC6 Protein

**Figure 1:**
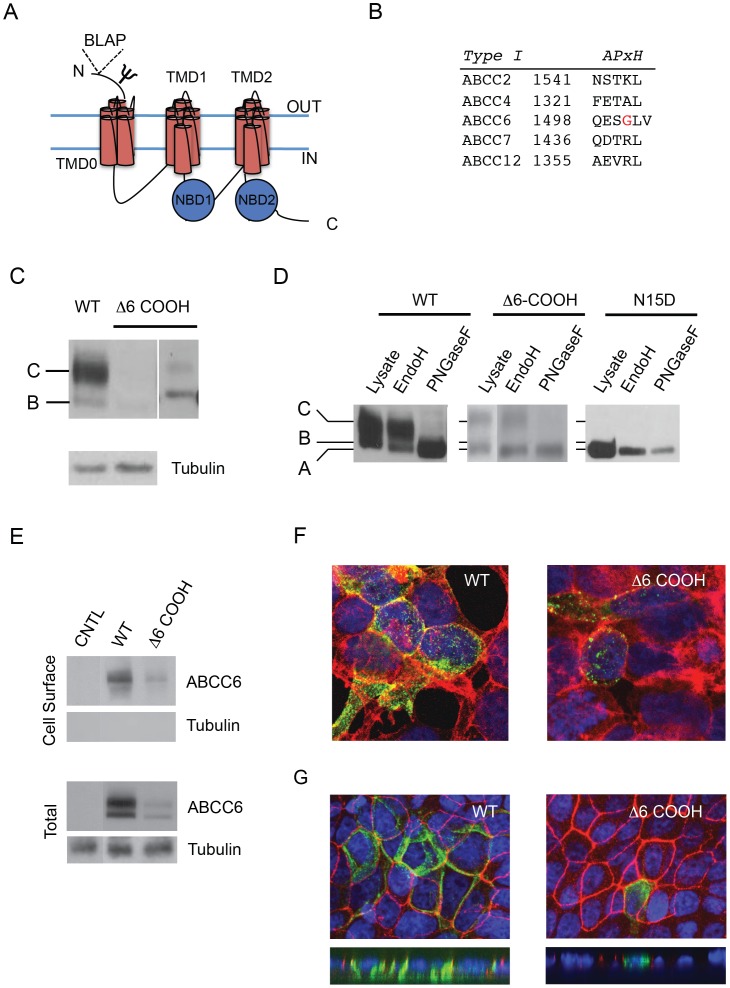
Alteration of ABCC6 trafficking by the PDZ-like C-terminus. To evaluate the potential role of the PDZ-like sequences at the C-terminus of ABCC6, wildtype and mutant proteins were expressed in HEK293 cells and evaluated by western blotting and immunofluorescence. *A,* a cartoon illustrating the domain organization and topology of ABCC6 is shown. ABCC6 contains three transmembrane domains and two nucleotide-binding domains. The single glycosylation site is represented as Ψ in the extracellular N-terminus. The insertion site for the biotin ligase acceptor peptide is also shown in the N-terminus at proline 4. *B,* a sequence alignment of known PDZ-containing ABCC family members is shown. The Type I consensus is shown above the alignment as is represented as: A, acidic; P, polar, X, any; and H, hydrophobic amino acids. The PXE-associate G1501S site is highlighted in red. *C,* representative western blots of the wildtype and Δ6-COOH ABCC6 proteins are shown after expression in HEK293 cells. The core and complexly glycosylated species are indicated on the left by B and C, respectively. Two exposures are shown for the Δ6-COOH to illustrate the formation of both band B and C at low levels in the mutant protein. *D,* endoglycosidase assays confirm the glycosylation state and differential electrophoretic migration of the ABCC6 proteins. The differential digestion of the band C protein by EndoH and PNGaseF demonstrates complex glycosylation, consistent with trafficking through the Golgi. The N15D substitution blocks N-linked glycosylation and is a reference for the unglycosylated wildtype and Δ6-COOH proteins. *E*, representative western blots from cell surface biotinylation experiments are shown. Cell surface expression is shown for cells mock transfected (CNTL) or transfected with wildtype or Δ6-COOH ABCC6, *Cell Surface*. Whole cell lysates are shown, *Total*, from samples prior to streptavidin pull-down. The control samples are taken from non-adjacent wells on a single gel/film. *F,* immunofluorescence images of the wildtype and Δ6-COOH proteins are shown. The ABCC6 proteins are shown in green, phalloidin is shown in red, and DAPI is shown in blue. Colocalization of the ABCC6 protein with phalloidin is consistent with ABCC6 trafficking to the cell surface in the wildtype protein and is decreased by the Δ6-COOH mutant. *G,* immunofluorescence images of the wildtype and Δ6-COOH ABCC6 proteins are shown after expression in polarized MDCK cells. The wildtype ABCC6 protein localizes to the basolateral membrane in polarized MDCK cells, *left.* The Δ6-COOH protein shows significant intracellular staining and a loss of basolateral targeting in MDCK cells, *right*. Both X–Y, *top*, and X–Z, *bottom*, images are shown. For *G*, ABCC6 is stained in *green* and ZO1 is shown in *red*. Western blots are representative of samples from at least three independent experiments.

ABCC6 is a member of the ABC-transporter family of proteins and is composed of five domains (Figure 1A). [34] Three transmembrane domains and two cytosolic, nucleotide-binding domains are encoded by a single polypeptide and putatively form the functional unit for transport. The two core transmembrane domains (TMD1 and TMD2) putatively provide the channel for substrate movement across the membrane. In addition an accessory transmembrane domain (TMD0) is located at the N-terminus. The function of this domain is unknown. Two nucleotide-binding domains are located intracellularly and are associated with ATP binding and hydrolysis, providing the energy for substrate transport.

A growing number of disease-associated mutations have been identified in the open reading frame of ABCC6. [5], [6], [35]–[37] Sequence analysis of the C-terminus shows a high degree of similarity to the PDZ ligand sequence identified in other ABCC family members (Figure 1B). These sequences have been shown to regulate subcellular localization, membrane stability, membrane motility and protein-protein interactions in other ABCC family members. [23]–[25], [27] The PDZ-like sequence in ABCC6 is highly conserved across species and includes the canonical Type I PDZ motif (acidic-polar-X-hydrophobic) found in closely related proteins and a C-terminal valine. [25], [38] The presence of this Val would putatively alter the binding of the PDZ ligand with its cognate partner in standard Type I PDZ ligand-domain interactions. However, multiple binding modes have been described structurally and diverge from the classical Type I binding. It is possible that the C-terminal valine would be accommodated in other binding modes with PDZ-domain containing proteins. [39], [40].

To investigate the role of this PDZ-like sequence in the regulation of ABCC6 biosynthesis and trafficking, the C-terminal sequence was deleted by PCR-based site directed mutagenesis and protein trafficking was assessed by western blotting after expression in HEK293 cells. The wildtype protein expressed robustly and was detected as two distinct bands by western blotting resulting from changes in glycosylation occurring in the ER and Golgi (Figure 1C, D). Under steady state conditions, the lower molecular weight species, band B, was found to be the core glycosylated, ER form of the protein. The higher molecular weight band was insensitive to EndoH but sensitive to PNGaseF digestion, consistent with protein modification by complex glycosylation in the Golgi. To further confirm these species were the result of changes in glycosylation, the single glycosylation site was mutated (N15D) and expressed. Digestion with both EndoH and PNGaseF resulted in no changes in electrophoretic mobility. Migration of the single N15D protein band was consistent with the fully deglycosylated protein, band A, in the wildtype ABCC6 expressing cells.

Deletion of the six C-terminal amino acids (Δ6-COOH), which includes the complete PDZ ligands found in other ABCC family members, showed dramatic changes in both protein biosynthesis and protein trafficking as measured by western blotting (Figure 1C, D). Steady state Δ6-COOH protein levels were reduced relative to the wildtype protein (Figure 1C). Both band B and band C species were reduced relative to wildtype. In addition, the quantity of band C protein was significantly decreased relative to the quantity of the band B form, consistent with either rapid turnover of the fully glycosylated protein or a decrease in biosynthetic efficiency and trafficking.

Cell surface biotinylation was used to assess the plasma membrane expression of ABCC6. Cell surface biotinylation of cells expressing the wildtype ABCC6 showed the presence of protein at the surface of HEK293 cells (Figure 1E). The protein appeared as a single band, consistent with the trafficking of the complexly glycosylated ABCC6 to the plasma membrane. Similarly, the Δ6-COOH ABCC6 protein also showed cell surface localization by biotinylation. As with the analysis of total steady-state protein levels, the quantity of cell surface Δ6-COOH ABCC6 was reduced when compared to the wildtype protein. Control cells, which were mock transfected, showed no detectable production of ABCC6 in both the total and cell surface fractions. Tubulin was present only in the total lysate fractions, consistent with its intracellular localization and protection from the biotinylation reaction.

Immunofluorescence was used to verify the subcellular localization of these proteins. The wildtype protein showed robust and diffuse expression in HEK293 cells, consistent with its trafficking through the secretory pathway and to the membrane (Figure 1F). Membrane localization was confirmed by colocalization with phalloidin. In contrast, the Δ6-COOH protein showed decreases in the number of stainable cells and the fluorescence intensity in cells expressing the protein (Figure 1F). Further, cells expressing the Δ6-COOH protein showed predominantly intracellular fluorescence, consistent with retention of the mutant in the ER seen by western blotting. Colocalization studies with BiP indicated the mutant protein was localized predominantly to the ER (data not shown). These data were consistent with the changes in steady state signal seen by western blotting of the Δ6-COOH protein and the reduction in complexly glycosylated species. No observable changes in immunofluorescence were seen between the wildtype and N15D glycosylation mutant (data not shown).

Finally, to assess trafficking in polarized cells, the wildtype and Δ6-COOH proteins were expressed in MDCK cells and assessed by immunofluorescence. Previous reports of wildtype ABCC6 trafficking indicate the ABCC6 protein is targeted to the basolateral membrane. [41] Consistent with this, the wildtype ABCC6 showed preferential targeting to the basolateral membrane in the MDCK cells (Figure 1G). Polarization of the MDCK monolayer was confirmed by staining with ZO1. Deletion of the PDZ-like sequence resulted in decreased targeting of the ABCC6 protein to the basolateral membrane and an increase intracellular protein. The predominant population of the Δ6-COOH protein colocalized with BiP, consistent with its accumulation in the ER and the immunofluorescence seen in HEK293 cells (data not shown).

### NBD2 Structure

**Figure 2:**
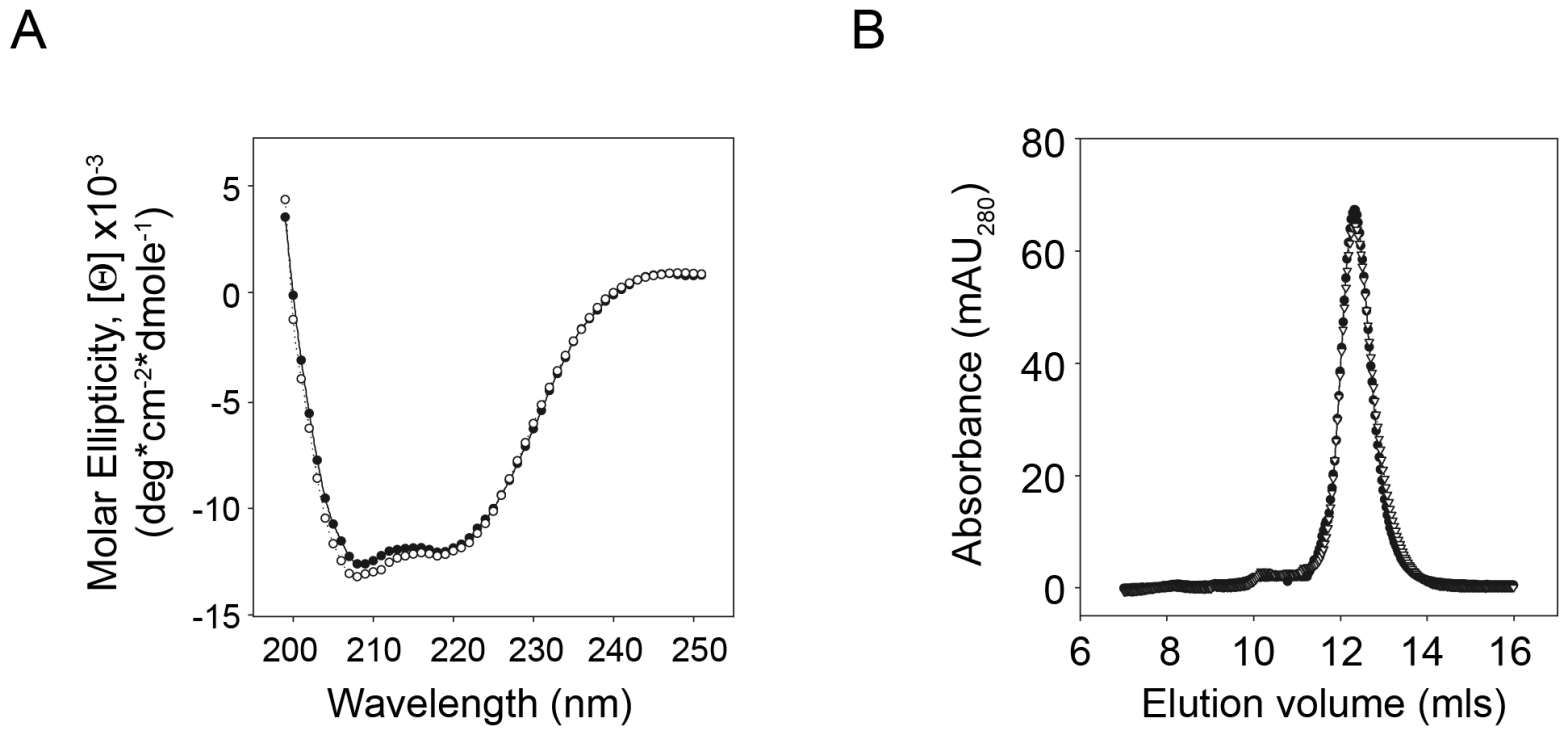
Structural characterization of wildtype and Δ6-COOH NBD2. To evaluate potential changes in ABCC6 NBD2 resulting from the C-terminal deletion, NBD2 was expressed and purified for *in vitro* analysis. *A,* CD spectroscopy was used to evaluate changes in the secondary structure of the mutant NBD2. Spectra were collected from 260 to 198 nm and corrected for buffer absorbance. The traces were smoothed using a window of 5 nm. The wildtype NBD2, *black circles*, shows a mixed α/β secondary structure qualitatively consistent with known structures of NBD proteins. The Δ6-COOH mutant NBD2, *open circles*, shows no significant differences in CD spectra. *B,* analytical gel filtration was used to evaluate changes in hydrodynamic radii of the wildtype and mutant NBD2 proteins. The wildtype protein eluted as a single symmetrical peak at ∼12.2 mls, consistent with a protein of ∼25,000 Da MW. The mutant proteins eluted similarly, with a peak at 12.2 mls. No discernible differences in either CD or GFC could be detected between the wildtype and mutant proteins.

Based on comparisons with the structures of other NBD proteins, the PDZ-like sequence is located at the C-terminus of the second NBD in ABCC6 and is not predicted to contribute to the core of the NBD fold. [31], [32], [42] To evaluate the possibility that altered NBD structure resulted from the Δ6-COOH deletion, the NBD2 domain was expressed and purified from *E. coli* for *in vitro* structural analyses. The wildtype and mutant NBD2 proteins expressed robustly and were purified using Ni-NTA and gel filtration chromatography. Changes in secondary structure of the purified NBD2 proteins were first evaluated using CD spectroscopy. The wildtype NBD2 proteins produced CD spectra consistent with mixed α/β-secondary structure (Figure 2A). These spectra were qualitatively consistent with those of solved crystal structures of other NBD proteins. [43] The Δ6-COOH NBD2 protein spectra showed no significant deviation from those of the wildtype NBD2 proteins, consistent with its folding to a similar mixed α/β structure. To evaluate the tertiary structure of the wildtype and mutant NBD2 proteins, hydrodynamic radii were measured using analytical GFC (Figure 2B). The wildtype protein eluted as a single symmetrical peak, consistent with a protein of ∼25 kDa molecular weight. The elution chromatographs showed no significant protein in either the column void or outside of the monomer peak, consistent with a highly purified and homogeneous sample. Elution volumes were calibrated using GFC standards to confirm apparent molecular weights. The mutant NBD2 protein eluted with a symmetrical profile similar to that of the wildtype NBD2. No significant protein was seen in the column void or outside of a single predominant peak corresponding to the monomeric NBD.

### Low Temperature Rescue of the Truncated ABCC6 Protein

**Figure 3:**
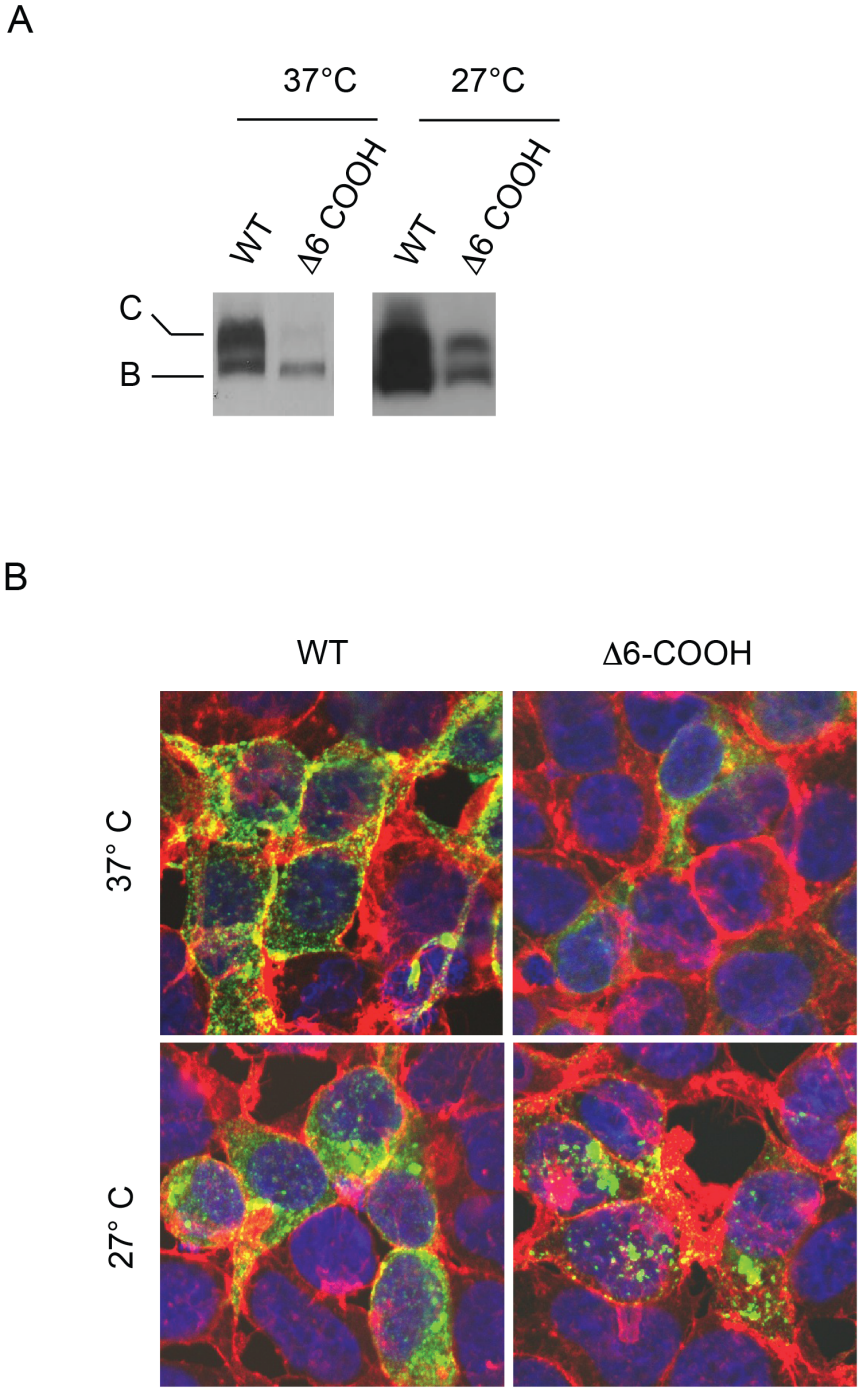
Rescue of ABCC6 trafficking by low temperature expression. Low temperature expression was used to further evaluate ABCC6 trafficking in HEK293 cells. *A,* western blots showing the expression of the wildtype and Δ6-COOH ABCC6 proteins at 37°C and 27°C. Expression of the wildtype protein at 27°C results in an increase in relative quantities of the core glycosylated, band B protein. Expressing the mutant protein at low temperature resulted in an increase in complexly glycosylated protein (band C), consistent with an increase in the formation or stabilization of this protein. *B,* indirect immunofluorescence of ABCC6 confirming the trafficking of the wildtype and mutant proteins is shown. Consistent with the western blotting, expression at low temperature results in redistribution of the mutant protein towards the plasma membrane. ABCC6 is shown in green, phalloidin is shown in red and DAPI is shown in blue.

To further elucidate the effects of the Δ6-COOH mutant, the ABCC6 proteins were expressed in HEK293 cells at low temperature. Previous studies of multiple membrane proteins suggest that biosynthetic and localization defects can often be corrected by expression at sub-physiological temperatures. [44]–[47] Expression of the wildtype and mutant ABCC6 proteins was performed at 27°C for 72–96 hours. Western blotting of the wildtype and mutant ABCC6 proteins showed dramatic changes in expression at low temperature. The wildtype protein expressed robustly, though changes in the relative levels of band B and Band C were observed (Figure 3A). Specifically, the quantity of band B protein was consistently increased when compared to the quantity of band C wildtype protein. This was consistent with an accumulation of the ER-associated form of the protein. In addition, the mutant protein showed an increase in both band B and band C forms of the protein (Figure 3A). The increase in expression was seen as both an increase in total steady state protein and an accumulation of the upper molecular weight, band C species.

The accumulation of the band C form of the protein was consistent with an increase in protein trafficking or a stabilization of the mature form of the protein in post-ER compartments. Immunofluorescence revealed similar changes in subcellular localization of the wildtype and mutant ABCC6 proteins (Figure 3B). The wildtype protein showed subtle increases in the quantities of intracellular protein by immunofluorescence. More strikingly, the mutant protein showed an increase in total ABCC6 protein staining. In addition, the Δ6-COOH protein appeared more diffuse and colocalization with membrane markers was increased after expression at 27°C. These data were consistent with an increase in the mutant protein at the cell surface.

### C-terminal Regulation of ABCC6 Degradation

**Figure 4:**
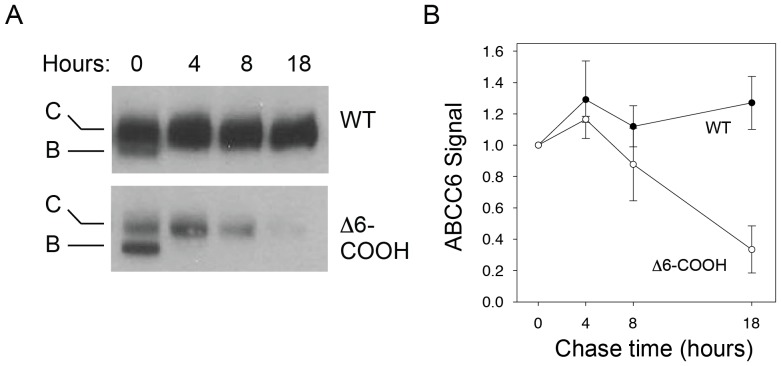
Impact of the C-terminus on ABCC6 turnover. Protein turnover was evaluated by cyclohexamide chase experiments after expression in HEK293 cells. *A,* western blots of the wildtype and Δ6-COOH proteins are shown after 0, 4, 8, and 18 hours of cyclohexamide treatment. The wildtype protein shows minimal changes after 18 hours of cyclohexamide treatment, while the mutant is decreased by ∼80% over this timecourse. The loss of band B protein in the mutant is consistent with the inhibition of new ABCC6 synthesis over the timecourse of treatment resulting from cyclohexamide treatment. *B,* summary data for cyclohexamide chase experiments are shown. Between 8 and 18 hours of cyclohexamide chase, the Δ6-COOH mutant protein is diminished by ∼80% relative to the wildtype protein. Data shown are mean +/− standard deviation from n = 3 experiments.

The changes in steady state protein levels were consistent with an increase in protein turnover associated with the Δ6-COOH mutation. To further evaluate how changes in protein turnover may be regulated by the PDZ-like sequence, cyclohexamide chase experiments were performed on HEK293 cells expressing ABCC6. Cells expressing either the wildtype or mutant protein were harvested at specific timepoints after cyclohexamide addition and evaluated by western blotting (Figure 4A). Cyclohexamide addition had little effect on the synthesis of either the wildtype or Δ6-COOH protein as judged by the relative band C - band B ratio at the zero hour timepoint. Wildtype band C protein levels appeared unaltered across the 18-hour timecourse of cyclohexamide treatment as no significant decrease in band C was seen by western blotting (Figure 4B).

In contrast, the Δ6-COOH protein showed increased protein turnover during the cyclohexamide treatment timecourse. At the zero timepoint, both band B and band C protein were observed for the Δ6-COOH protein (Figure 4A). The band B protein did not appear in subsequent timepoints, consistent with either its maturation to the band C form or its degradation from the ER. However, the band C Δ6-COOH protein showed increased degradation across the 18-hour timecourse of cyclohexamide treatment (Figure 4B). At eight hours, the Δ6-COOH protein showed a ∼20% decrease in band C, as compared to the zero hour timepoint. At 18 hours, the quantity of band C Δ6-COOH protein was reduced by greater than 80%, as compared to the zero hour timepoint. The increase in protein turnover suggested that increased rates of degradation of the Δ6-COOH protein contributed to the changes in expression seen by western blotting and immunofluorescence under steady state conditions.

### Plasma Membrane Turnover of ABCC6

To further evaluate changes ABCC6 dynamics, a biotin ligase acceptor peptide (BLAP) was introduced into the N-terminus of the ABCC6 proteins (Figure 1A). Previous studies have shown that the BLAP tag is selectively modified by the BirA biotin ligase and can be used to efficiently label proteins that include this specific peptide sequence. [48], [49] To accomplish labeling, the ABCC6-BLAP proteins were co-expressed with BirA that has been fused with an ER-localization (KDEL) sequence or labeled with purified BirA at the cell surface. When both the ligase and tagged proteins are co-expressed, the nascent polypeptide is biotinylated by the BirA ligase during biosynthesis and ER membrane integration. The cell surface biotinylated protein can then be labeled by addition of membrane impermeant, fluorescently-conjugated streptavidin.

**Figure 5:**
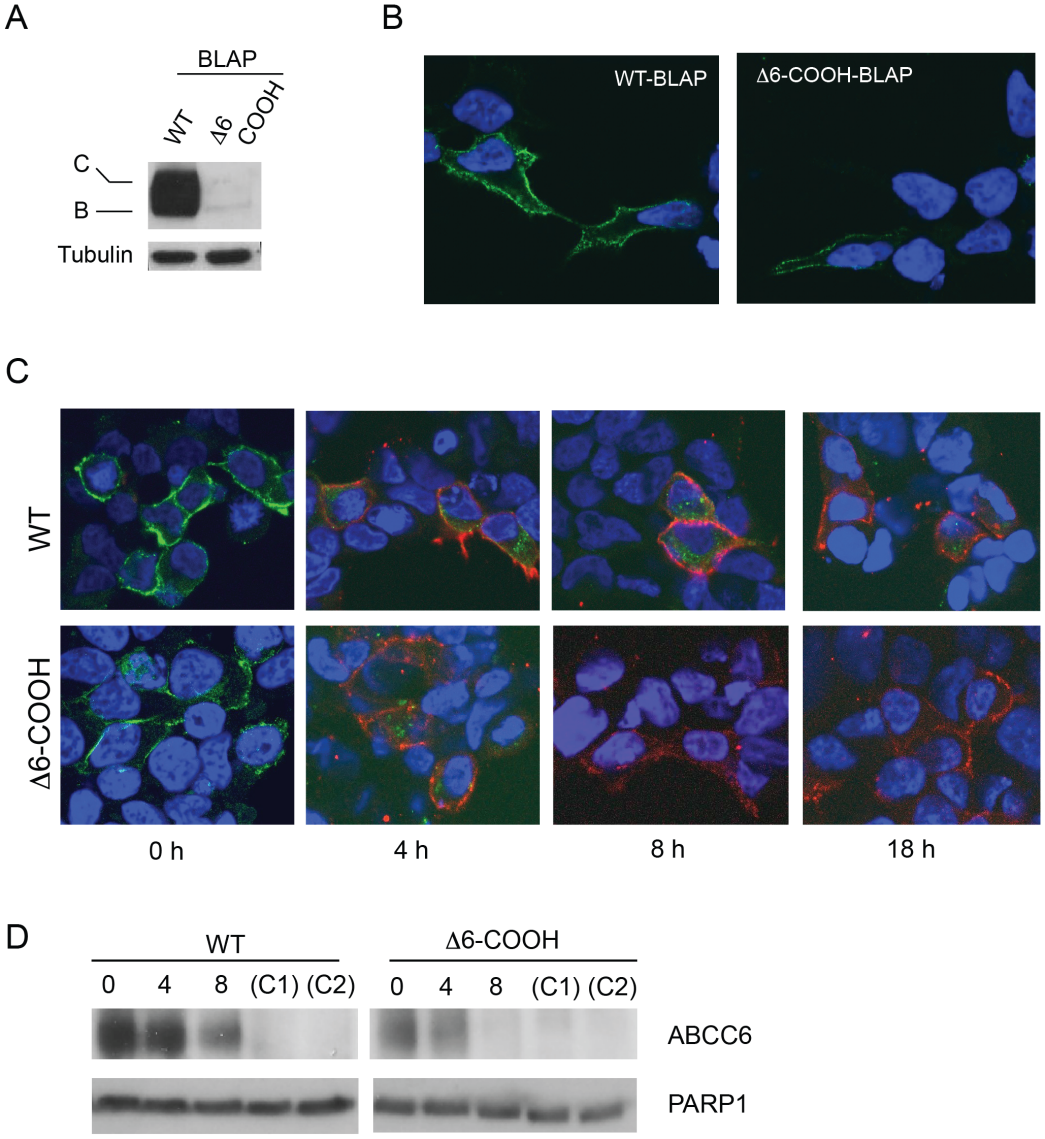
Regulation of cell surface stability by the C-terminus. Cell surface stability was evaluated by selective biotinylation of the ABCC6 protein using the BirA ligase and acceptor peptide in the extracellular N-terminus (see Figure 1A). *A,* western blots of the wildtype and Δ6-COOH ABCC6 protein with the BLAP tag are shown. The inclusion of the BLAP tag in the N-terminus had no detectable effect on the trafficking of the wildtype or mutant ABCC6 proteins evaluated by western blotting. *B,* fluorophore-conjugated streptavidin was applied to the culture media and cell surface expression of the BLAP ABCC6 protein was evaluated by fluorescence microscopy. Consistent with western blotting, no detectable differences were seen between the BLAP tagged and untagged ABCC6 proteins. The wildtype protein expressed robustly in HEK 293 cells, while the mutant protein was only labeled in a small fraction of cells transfected. *C,* fluorescence analysis of the timecourse of ABCC6 internalization and degradation from the cell surface is shown. The BLAP tagged proteins were sequentially labeled with fluorophore-conjugated streptavidin. Initial staining, time zero, was performed using AlexaFluor-488, *green*, and secondary labeling was performed using AlexaFluor-555, *red.* The internalization and degradation of ABCC6 could be seen over the course of 4–18 hours as the loss of green signal. *D,* western blots of cell surface labeled ABCC6 are shown. Streptavidin was incubated extracellularly on intact HEK293 and the BLAP-tagged ABCC6 protein was bound and washed. The lystes were subjected to SDS-PAGE and western blotting. The conjugated ABCC6-streptavidin complex could be distinguished readily from the total ABCC6 protein, allowing for the evaluation of plasma-membrane ABCC6 protein. Proteins were labeled, washed and incubated for zero to eight hours before lysis. Negative controls included expression of the BLAP-ABCC6 protein without streptavidin treatment (C1) and mock-transfected HEK293 cells treated with BirA and streptavidin (C2). Both negative controls showed no staining, consistent with specific detection of labeled BLAP-tagged ABCC6 in the experimental samples.

Steady-state expression of the wildtype and Δ6-COOH BLAP proteins was assessed by western blotting and immunofluorescence. As seen with the untagged ABCC6 proteins, the wildtype protein trafficked with high efficiency through the secretory pathway. Western blotting confirmed strong band C expression of the BLAP tagged wildtype ABCC6 (Figure 5A). The Δ6-COOH BLAP ABCC6 showed a dramatic reduction in total expression compared to wildtype. In addition, the relative quantities of band C and band B were altered in the Δ6-COOH mutant when compared with wildtype. These changes were consistent with those seen in the untagged ABCC6 proteins (Figure 1). Cell surface detection of the protein after co-expression with KDEL-BirA, visualized by extracellular application of an AlexaFluor conjugated streptavidin, confirmed the cell surface expression of the wildtype protein (Figure 5B). As with the biotinylation and immunofluorescence of the untagged Δ6-COOH ABCC6 protein, the BLAP tagged mutant showed reduced cell surface expression with fewer cells and lower fluorescence signal evident for the mutant. These results suggested the presence of the BLAP tag had no discernible effect on the behavior of the wildtype and mutant ABCC6 proteins.

Using this system, we assessed the lifetime of the plasma membrane resident BLAP-ABCC6 proteins using both fluorescence and western blotting of the ABCC6-biotin-streptavidin complex, as previously described. [49] Pulse-chase cell surface labeling of the wildtype and Δ6-COOH proteins was accomplished by sequential labeling using two AlexaFluor-conjugated streptavidin proteins. Cells were labeled on ice with an AlexaFluor-488 conjugated streptavidin and the excess streptavidin was removed by washing with PBS. The initial labeling corresponded to the cell surface “pulse,” selectively tagging the plasma membrane resident ABCC6 protein. The cells were then incubated for designated periods – the “chase” - at 37°C before being labeled using an AlexaFluor-555 conjugated streptavidin, washed and fixed for visualization. The second labeling facilitated the identification of the cell surface and demonstrated continued expression and trafficking of ABCC6 during the experimental timecourse.

The wildtype ABCC6 protein showed robust cell-surface labeling (Figure 5C) at the initial timepoint (0 hours) with the AlexaFluor-488-conjugated streptavidin. The inability to detect significant AlexaFluor-555 label at the zero timepoint suggested that the vast majority of plasma membrane ABCC6 was bound by the AlexaFluor-488-conjugated streptavidin. At four, eight and 18 hours after the AlexaFluor-488 “pulse,” the wildtype protein was partially endocytosed, as evidenced by the internalization of the AlexaFluor-488 label. This relocalization was confirmed by the visualization of AlexaFluor-555 labeled ABCC6 at the cell surface. By 18 hours the majority of plasma membrane ABCC6 initially labeled at the zero time point appeared to have been internalized and/or degraded. The loss of fluorescence after internalization likely resulted from both dilution of the fluorophore from the plasma membrane into multiple intracellular compartments and degradation of the streptavidin conjugate.

In contrast, the Δ6-COOH protein showed increased internalization and degradation relative to the wildtype protein (Figure 5C). Cell surface labeling of the mutant protein was less robust than the wildtype at the initial timepoint. Additionally, by 4 hours the majority of mutant ABCC6 protein had been internalized and degraded, as evidenced by a loss of green fluorescence. By eight and 18 hours, the ABCC6 protein labeled at the initial timepoint was undetectable. As with the wildtype protein, Δ6-COOH ABCC6 streptavidin-conjugated AlexaFluor-555 labeling after the “chase” period facilitated identification of the cell surface and demonstrated continued expression and trafficking of the ABCC6 protein.

To verify the turnover of these cell surface ABCC6 proteins biochemically, western blots of the streptavidin-bound BLAP-ABCC6 protein were evaluated. Previous studies have shown that the streptavidin-biotin association is resistant to SDS, with the biotin and streptavidin remaining complexed after SDS-PAGE analysis. [49] The resulting size shift, associated with the ABCC6-streptavidin complex, effectively separates the labeled and unlabeled proteins when separated by PAGE. Thus, the cell surface pool of ABCC6 can be analyzed independent of the internal pools of protein. Cells were labeled with streptavidin and incubated for up to eight hours. Cells were harvested at specific timepoints and the lysates were subjected to SDS-PAGE and western blotting (Figure 5D). The wildtype protein showed strong expression at the initial timepoint and appeared to decay after approximately 4 hours. At eight hours, the wildtype protein was reduced, but still readily detectable. The mutant protein showed a decrease in total expression and an increase in the rate of degradation. By four hours, the mutant protein showed significant reduction in signal and was undetectable at eight hours. The intracellular protein PARP-1 (110 kDa) was used as a loading control to facilitate simultaneous detection of the streptavidin-ABCC6 complex and the control.

### Mechanisms of ABCC6 Degradation

**Figure 6:**
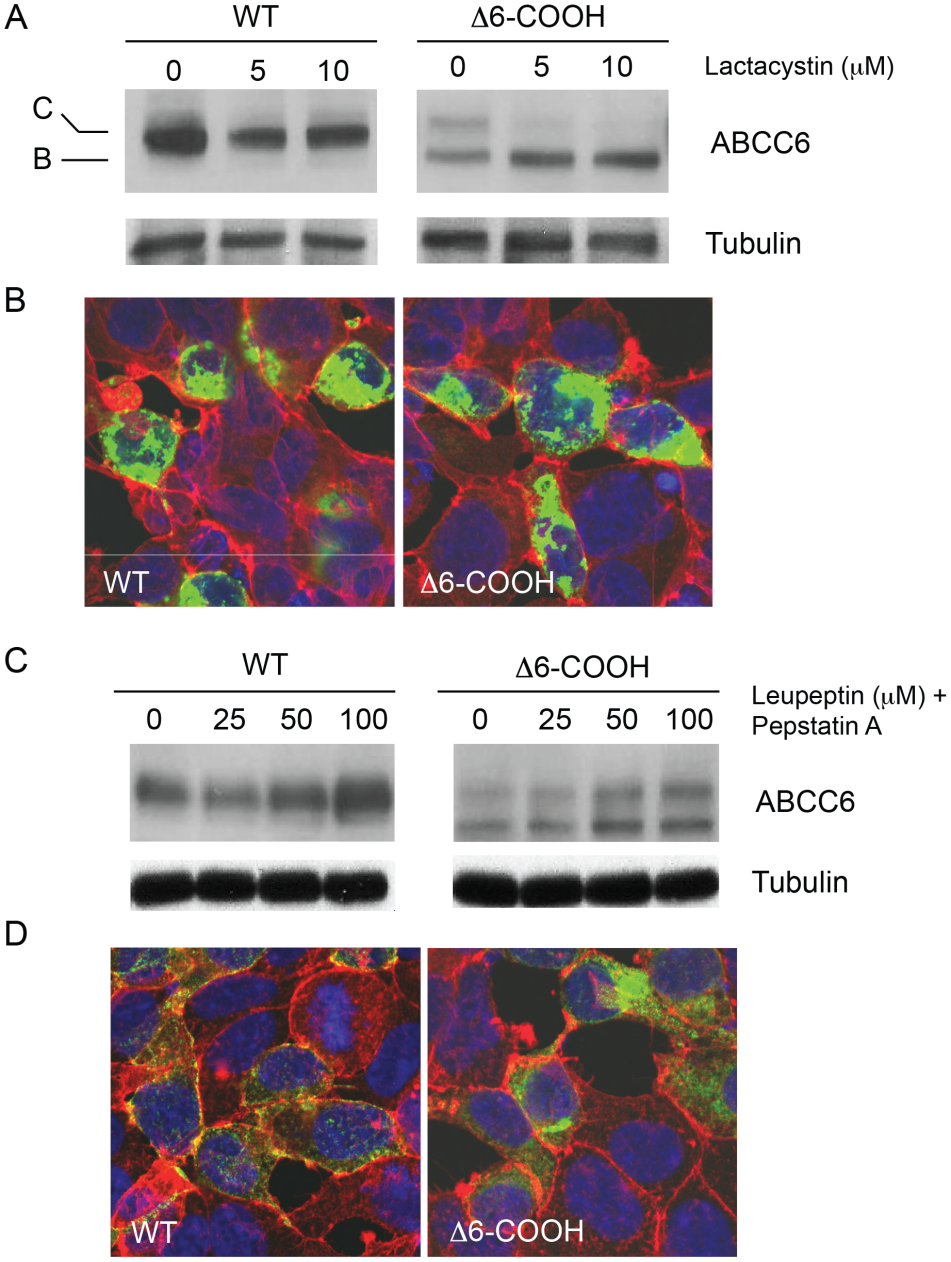
Impact of the C-terminus on ABCC6 degradation. ABCC6 degradation was evaluated after treatment with a proteasome inhibitor (lactacystin) or a combination of lysosomal protease inhibitors, (leupeptin and pepstatin) and assessed by western blotting. *A,B,* inhibition of the proteasome by lactacystin results in an accumulation of the ER-resident, band B protein in the mutant ABCC6. *A,* increasing lactacystin concentrations from 0–10 µM, results in an accumulation of the band B form of the mutant ABCC6 protein as seen by western blotting. No increase in the formation of the band C, complexly glycosylated protein is seen for either wildtype or mutant ABCC6 with lactacystin treatment. *B,* immunofluorescence of the ABCC6 proteins reveals the wildtype and mutant accumulate after proteasome inhibition, but the mutant fails to redistribute to the cell surface. ABCC6 is shown in green, phalloidin is shown in red and DAPI is shown in blue. *C,D,* lysosomal inhibition results in an increase in the complexly glycosylated, band C protein for both wildtype and mutant ABCC6. *C,* a dose response of leupeptin/pepstatin treatment is shown from 0–100 µM leupeptin treatment in the presence of 1 µg/ml pepstatin. Increasing pepstatin concentrations resulted in an increase in the band C ABCC6 protein. *D,* immunofluorescence of HEK293 cells treated with leupeptin/pepstatin is shown. Treatment with leupeptin/pepstatin resulted in an increase in the quantities of ABCC6 intracellularly. ABCC6 is shown in green, phalloidin is shown in red and DAPI is shown in blue.

To further evaluate the mechanisms associated with the Δ6-COOH turnover and determine where in the cell this turnover occurs, lactacystin and leupeptin/pepstatin were used to probe cellular degradation pathways. Lactacystin was utilized to block the proteasome, which is the principle degradation machine associated with ER-associated degradation (ERAD). Inhibition of the proteasome by lactacystin treatment showed minimal effects on the maturation of the wildtype protein, as evaluated by western blotting (Figure 6A). Subtle changes in band C quantities were seen after treatment with lactacystin. No significant accumulation of band B protein was seen in cells expressing the wildtype protein, consistent with its trafficking from the ER to the Golgi. In contrast, the band B Δ6-COOH protein accumulated with lactacystin treatment, consistent with a reduction in ERAD. No significant changes in band C protein were seen with lactacystin treatment for the Δ6-COOH protein. The accumulation of band B and lack of band C formation suggested that inhibiting the proteasome did not facilitate trafficking of the mutant protein to the Golgi and post-Golgi compartments. Immunofluorescence confirmed the accumulation of intracellular Δ6-COOH protein (Figure 6B).

To evaluate protein turnover from the cell surface, a combination of leupeptin and pepstatin were utilized to block lysosomal degradation. Treatment with leupeptin/pepstatin resulted in an increase in band C protein for both the wildtype and Δ6-COOH under steady state conditions (Figure 6C). In a dose-dependent manner, increasing leupetin doses resulted in increasing band C protein levels. Band B protein levels for the Δ6-COOH protein appeared unaffected by these treatments, consistent with its degradation by the proteasome seen in Figure 6A. The increased accumulation of band C Δ6-COOH protein suggested that a population of this protein trafficked out of the ER and into post-Golgi compartments, where it was degraded in the lysosome. Similarly, when evaluated by immunofluorescence, both wildtype and Δ6-COOH showed increased intracellular accumulation (Figure 6D). The accumulation of intracellular protein seen by immunofluorescence was consistent with western blots of ABCC6 and demonstrated that the post-Golgi pool of ABCC6 was degraded by the lysosome.

## Discussion

The regulation of ABC transporter trafficking and activity is complex, requiring multiple folding and assembly steps to form the functional transporter. [50]–[52] In addition to the biosynthetic processes, ABC transporter localization and stability are regulated by multiple protein sequences. [24], [38], [53], [54] Among these, PDZ ligands have been shown to contribute to protein localization and stability at the plasma membrane. [29], [55]–[57] Given the apparent similarity of the ABCC6 C-terminal sequence to other PDZ-containing ABCC transporters and the presence of a disease-associated mutation within this region, we chose to evaluate the role of this sequence in the expression and regulation of ABCC6.

Previous studies have demonstrated that the C-terminal sequences in other ABC transporters play critical roles in the trafficking, localization and function of these proteins. [55]–[58] Similarly, the deletion of the PDZ-like sequence in ABCC6 suggested that this region of the protein also contributed significantly to the biosynthesis and trafficking of the protein. Removal of the C-terminal six amino acids, predicted to contain the residues required for a PDZ-domain interaction, resulted in decreased steady state protein levels and changes in protein localization in both polarized and non-polarized cells (Figure 1). These changes appeared to decrease total protein expression resulting from an increase in protein degradation (Figure 4, 5). The changes in degradation were likely the result of increased protein turnover in the ER and at the plasma membrane, as the core glycosylated protein and the complexly glycosylated protein were reduced by the C-terminal truncation. Both forms could be alternately stabilized by protease inhibition. The ER associated form was most strongly stabilized by inhibition of the proteasome while the post-ER pool of protein was most strongly stabilized by inhibition of the lysosome (Figure 5). These changes in protein degradation did not appear to be caused by gross changes in the local structure of the NBD, as purification of the wildtype and mutant NBD2 were similar. The local structural properties assessed spectrophotometrically and hydrodynamically were also similar for both NBDs (Figure 2). Additional work is required to assess the possibility that global changes might be propagated into the ABCC6 structure through altered domain-domain contacts. However, these data are consistent with previous reports of regulated ABC transporter trafficking by PDZ associations in the secretory pathway. [54], [59].

Previous studies have shown that PDZ sequences contribute to the regulated trafficking of other proteins, including ABC transporters, though the exact roles of these sequences vary by protein. In CFTR (ABCC7) the PDZ sequences regulate protein localization and stability in the plasma membrane, with little influence on the biosynthetic processing or trafficking of the protein. [27], [38] In contrast, removal of the PDZ sequence from ABCC2 (cMOAT, MRP2) can alter either localization to the apical membrane or changes in protein trafficking through the biosynthetic pathway. [57], [58] Further, these processes may be regulated by phosphorylation of residues within the PDZ sequence, providing a cellular mechanism to dynamically regulate these associations. [23] Similar effects on trafficking and localization have been reported for NMDA receptors, K^+^-channels, and aquaporin channels, among others. [20], [55], [56] These results are consistent with models wherein PDZ ligands are critical regulators of intracellular protein-protein interactions along the secretory and endocytic pathways as well as protein localization anchors at the plasma membrane. [24].

Whether this sequence serves as a *bona fide* PDZ sequence that binds a PDZ-domain containing protein or serves as a target for other cellular proteins/processes is yet to be elucidated and deserves further investigation. However, a single PXE patient in which the G1501S mutation was found was reported to show phenotypic abnormalities in the eye (bleeding/scarring) and mild presentation in the skin (papules/bumps), but presented without apparent GI, vascular or cardiac symptoms. [37] It is possible that tissue specific changes in protein localization or protein-protein association may contribute to the phenotype associated with this specific mutation. Recent works suggests that functional ABCC6 at the plasma membrane is coupled to the secretion of nucleotides and the production of extracellular pyrophosphate. However, nucleotide secretion is not mediated directly by ABCC6, suggesting that plasma membrane ABCC6 function may be coupled physically or functionally to the activities of other transmembrane proteins. [60] This indirect regulation of secretory activities could be accomplished by specific protein-protein interactions mediated by the ABCC6 PDZ-like sequence at the plasma membrane.

Taken together, our data suggest that the C-terminal PDZ-like sequence is critical for the regulated trafficking and membrane localization of ABCC6. Though this sequence varies from the canonical PDZ-sequences found in other ABCC transporters, its removal results in decreased protein expression and increased degradation. These data provide evidence that the C-terminal sequences in ABCC6 serve to regulate its biosynthetic processing and membrane stability, thereby providing novel insight for the regulation of ABCC6 in normal physiology and mechanisms for its disruption in disease pathophysiology.
